# Mixed-methods to explore barriers to the use of food security initiatives in a historically black college and university (HBCU)

**DOI:** 10.1186/s12889-024-20627-1

**Published:** 2024-11-11

**Authors:** Janet Antwi, Yetunde Olawuyi, Modupe Ifafore, Innocent Opara

**Affiliations:** Department of Agriculture, Nutrition and Human Ecology, Prairie View A&M University, 100 University Dr, Prairie View, TX 77446 USA

**Keywords:** Food security, Food pantry, Barriers to campus resources, HBCU

## Abstract

**Background:**

To assess students’ use and perception of the initiatives to tackle food insecurity (FI) in a Texas historically black college and university (HBCU).

**Methods:**

HBCU students > 18 years (288 students). A mixed-methods approach involving an online survey, focus group discussions and key informant interviews were conducted among the students and major stakeholders to evaluate the prevalence of FI, and gain insights into the challenges to accessing, utilizing and effectively implementing food security (FS) initiatives.

**Results:**

FI affected 63.5% of the participants. The awareness and utilization of the food pantry were 27.9% and 8.1% respectively while for the meal share programs, awareness and utilization were 30.1% and 15.0%, respectively. A significant association was found between FI and the place of residence (*p* = 0.027). Binary logistic regression model showed that students’ school year was a predictor of food pantry awareness (*p* < 0.05), residence and FI status were predictors of the meal share program utilization (*p* < 0.05). Five themes, including poor awareness and visibility of campus resources, bureaucratic process and logistics, insufficient funds, personnel and other resources to run the initiatives, ineffective communication, and stigma due to location emerged as the main barriers to the use of the resources.

**Conclusions:**

This study emphasizes the necessity for HBCUs to address these hurdles in order to successfully help students in need with FI resources.

## Introduction

Food insecurity (FI) is characterized by limited access to nutritionally adequate, safe foods or uncertainty in acquiring socially acceptable foods on a consistent basis [1, 2]. It is a serious problem that many American college students deal with which can negatively affect their academic standing, general well-being, and long-term health [3, 4]. Studies have shown that FI impacts as many as 10–75% of college students, with higher percentages observed among students attending historically black colleges and universities (HBCUs), which predominantly serve minority student populations [4–7]. The difficulties of FI are common among college students because many of them are dealing with budgetary limits, growing tuition expenses, and conflicting goals that make it difficult to afford a steady, wholesome diet [3, 8]. Beyond just causing hunger, FI affects students’ academic performance, emotional and physical health, and overall well-being [4]. Studies have linked student food hardship to low educational outcomes. Food hungry students have poorer grade point averages [3, 7], take fewer classes, and are more likely to drop out of courses or institutions [8–11]. 

HBCUs were largely formed after the Civil War to satisfy Black Americans’ educational needs [8]. They play a crucial role in providing African American students with access to higher education, thereby facilitating their upward social mobility. They have a history of supporting students’ health and wellbeing in addition to their academic achievement. Approximately 270,000 students nationwide attend HBCUs; the majority are African Americans from low-income families [8]. These students encounter particular difficulties in obtaining FS on campus. Students at HBCUs face increased difficulties, including FI, housing poverty, and homelessness [8, 9]. According to available data, HBCU FI rates are either greater or comparable to those of other colleges and institutions [7, 10]. A research on student debt found that 44% of Black borrowers currently enrolled in HBCUs had to forgo meals due to insufficient funds for food [10]. Students facing basic necessities insecurity often have to prioritize their expenses, choosing between rent, food, and whether to attend online or in-class lectures. This insecurity not only affects their academic performance but also their overall well-being [8, 11]. Few HBCUs have developed extensive FS strategies to methodically address student needs, despite the high incidence of FI. According to a 2019 survey, fewer than half of HBCUs offered meal voucher programs, food pantries, or other assistance aimed at reducing student hunger [11]. Despite increased efforts in recent years, there are still major obstacles that prevent FS programs at HBCUs from being implemented effectively [12]. Although a number of FS programs, including meal assistance programs, campus-based food pantries, campus community gardens, and financial aid initiatives, have been implemented to address these issues, little is known about how successful these initiatives are at HBCUs as the rate of FI in HBCUs are still reported to be high. A recent study by Duke et al. [7] reported that nearly 3 in 4 students (72.9%) at HBCUs reported experiencing some level of FI in the past year, a rate significantly higher than those at predominantly White institutions (PWIs) with meal plan participation failing to prevent FI. The high prevalence of FI among HBCU students highlights the need for more in-depth research into their lived experiences to develop institution-level food policies that effectively support their academic success and overall well-being. FS programs must be culturally relevant and accessible to be effective. HBCUs often serve a diverse student body with unique cultural and dietary needs. Exploring barriers can help develop initiatives that are more culturally appropriate and thus more likely to be utilized by students [13]. Some of the barriers reported by previous studies include time issues, lack of transportation, limited food pantry hours of operation, and social stigma [13, 14]. Designing focused interventions and creating an atmosphere that supports student success require an understanding of the unique obstacles to the adoption and use of FS programs within the HBCU setting.

The Texas HBCU studied is a university which took some steps to combat the alarming trend of FI. An emergency resource center was founded by the HBCU to support the university’s aim of assisting students in overcoming obstacles that stand in the way of their academic pursuits and successful matriculation by offering them the tools they need to overcome poverty, homelessness, and FI. Fresh produce, meats, and nonperishable food products are available to all registered students at the university through the Reserve Market, subject to their eligibility for a Food Bank Food Scholarship- a program designed to address FI among college students by providing eligible students with access to food pantry services or meal plans on campus. These scholarships aim to ensure that students have consistent access to nutritious meals, allowing them to focus on their studies without worrying about where their next meal will come from [15]. In January 2022, the University also partnered with Swipe Out Hunger, a nationwide group dedicated to eradicating hunger among college students. A Meal Share Program (MSP) was launched through this partnership to support students in need of emergency aid. Nevertheless, no research has been done on how the programs are used or viewed by students. This research examined the obstacles that impede the efficient execution and application of FS programs in the distinct setting of an HBCU. The study’s objectives were to ascertain the prevalence of FI among students, primary obstacles impeding the effective implementation of FS programs in an HBCU, evaluate the degree of awareness and utilization of current FS programs among HBCU students, and obtain perspectives from key stakeholders, such as academic staff, administrative personnel, and students, regarding the perceived difficulties and possible remedies pertaining to FS on campus.

## Methods

### Participants and study design

A mixed-methods research with convergent parallel design was carried out in order to have a comprehensive understanding of the phenomena being researched through numerical data and nuanced contextual insights. We conducted a cross-sectional online survey open to all students at the Texas Historically Black College and University (HBCU) to assess the prevalence of FI and the awareness and utilization of available resources on campus. From April to June 2023, we sent recruitment emails to all University students, inviting them to participate in an online survey focusing on student FI. The survey was also advertised on flyers posted at designated locations and the flyers distributed to participants one-on-one. Students who are < 18 years, or who do not have access to online content through an electronic device were excluded from the study. Five hundred and seventy undergraduate and graduate students responded to the online survey but only 288 filled out the questions on FI. The survey was voluntary and anonymous.

Two 60-minute focus group discussions (FGDs) of 4 students each and 15 key informant interviews (KII) of 20–40 min each were conducted with major stakeholders to gain insights into the challenges to accessing, utilizing and effectively implementing the initiatives. The stakeholders interviewed were individuals who had direct dealings with the FS initiatives on campus and were purposively chosen. They included staff of the Health Service/food pantry, extension agents, community leaders, Cafeteria, staff and students of the student-led garden, the meal share program and student government association. The students who participated in the FGDs were those who were experiencing FI. Invitation was sent to all participants who were classified based on the FS questionnaire to participate in the FGD but only 8 of them responded. Participants received information about the study and a signed informed consent was acquired. The University’s Institutional Review Board approved the research.

### Data collection

Data were collected via Qualtrics, an online software. The survey contained a 97-item questionnaire but the main questions used for this study were sociodemographic characteristics, campus resources and FS questions. The FS questions were derived from the United States Department of Agriculture-(USDA) short form of the 12-month FS scale. The interview and FGD guide consisted of structured open-ended questions that were developed based on the objectives of the study and were designed to highlight critical issues, perspectives based on insights from literature. Participants were questioned regarding their opinions of the campus food environment, their involvement in various campus FS programs, and their personal experiences with eating healthily on campus. For example, in KII, participants were asked about the operations and impact of the Student-led community garden, food pantry, and Panther Meal Share plan to address FI on campus and the challenges in their implementation; how food choices of students affect their health; how to improve food options on campus and promote healthy eating habits among students; awareness, knowledge and use of local community resources to limit FI on campus; and describe what food is served and wasted in the cafeteria and whether students are meeting their dietary needs. The FGDs questions asked were on participants to share their experience on barriers to eating healthy on campus; what limit students’ access to obtaining fresh produce; how can students be made more aware of campus initiatives to address FI; what cultural beliefs and practices within the university influence attitudes and behaviors related to healthy living; what can be done to improve the availability and accessibility of fruits and vegetables on campus; and how can students be encouraged to take more fruits and vegetables. The guides were reviewed by members of the research team and the sessions were audio-recorded. The interviewers took notes on the respondents’ keywords and phrases and double-checked the validity of the data by repeating the response to ensure that the interviewers understood and interpreted what they had said and had it correctly documented.

### Analysis

The survey data were cleaned and analyzed using IBM SPSS Statistics for Windows, Version 28.0 (IBM Corp. Armonk, NY). The USDA’s scoring system was used for the six questions to assess FS status. Raw ratings between 0 and 1 suggest high/marginal FS, while raw values of 2–4 low FS, and 5–6 very low FS, where ‘low FS’ and ‘very low FS’ are FI measures [16]. Descriptive statistics were calculated for all demographic and campus resources questions. The continuous variable ‘age’ was recategorized into six groups. Missing data were excluded from the analysis. Cross-tabulations, coupled with Chi-square testing, were employed to explore associations between demographic factors and campus resources questions at a significance level of α 0.05. Four binary logistic regression models with awareness of food pantry, awareness of the meal share program, utilization of food pantry and utilization of the meal share program as the outcomes, were estimated to identify sociodemographic characteristics (predictor variables) associated with the dependent variables. Regression models were adjusted for all sociodemographic characteristics.

The interviews were transcribed into Microsoft Word, deidentified, and carefully reviewed for accuracy. Subsequently, they were imported into QSR NVivo 12 Pro (QSR International Pty Ltd. Release 1.0, 2020). A thematic analysis approach was employed, where the data were systematically coded and examined to identify overarching themes by two members of the research team. Codes were merged when they encapsulated similar statements. Text-search queries were utilized to identify instances where a theme had been addressed by a respondent elsewhere, and these statements were coded in parallel [17]. 

## Results

Table 1 shows that, out of a total of 288 students, most of the participants for the quantitative analysis were female (72.1%), aged 18–24 years (75.4%) and of Non-Hispanic Black/African American (83.5%) origin. Table 2 shows very few (27.9%) of the participants knew about the campus food pantry and just 8.1% had used it. Similarly, only 30.1% of the participants were aware of the meal share program with 15% having used it. The reasons cited for not using both the food pantry and the meal share program included lack of awareness, time, staying off-campus among others. However, the topmost reason for non-usage was lack of awareness with 79.2% and 90% of the participants being unaware of the food pantry and the meal share program respectively. FI levels were high overall, with 63.5% reporting low FS and very low FS.

**Table 1:** Sociodemographic characteristics of participants

Variables		Frequency *N* (%)
Gender	Male	77 (26.8)
	Female	207 (72.1)
	Non-binary (Specify below)	3 (1.0)
Age Category	18–21	156(55.7)
	22–25	62 (22.1)
	26–29	17(6.1)
	30–39	26 (9.3)
	40–49	15 (5.4)
	>=50	4 (1.4)
Mean Age/ Std deviation	24.10 ± 8.014	
Ethnic Background	Non-Hispanic Black/African American	238 (83.5)
	Hispanic/Latino	25 (8.8)
	Asian	5 (1.7)
	Native American/Other Pacific Islander	1 (0.4)
	Non-Hispanic White	7 (2.5)
	Other (specify)	9 (3.2)
Classification	Freshman	37 (13.0)
	Sophomore	73 (25.6)
	Junior	64 (22.5)
	Senior	66 (23.2)
	Graduate Student	45 (15.8)
College	College of Nursing	27 (10.5)
	College of Agriculture, Food and Natural Resources	18 (7.0)
	Brailsford College of Arts and Sciences	78 (30.4)
	College of Business	36 (14.0)
	College of Education	38 (14.8)
	College of Engineering	35 (13.6)
	College of Juvenile Justice	14 (5.4)
	School of Architecture	11 (4.3)
Marital Status	Married	16 (5.6)
	Single	263 (91.6)
	Divorced/Widowed	6 (2.1)
	Other	1 (0.7)
Work Status	Not working	129 (44.9)
	Work Part-time	104 (36.2)
	Work full time	40 (13.9)
	On disability	2 (0.7)
	Other, specify	12 (4.2)
Where do you live	Campus Hall Residence	150 (52.4)
	Off-Campus	99 (34.6)
	With family (parents, husband, wife, children, etc.)	37 (12.9)

**Table 2:** Campus resources and food insecurity characteristics

Variables		Frequency *N* (%)
Do you know if there is a food pantry on campus?	Yes	80 (27.9)
	No	207 (72.1)
Do you go to the food pantry to pick up food?	Yes	23 (8.1)
	No	262 (91.0)
Reason for non-usage of food pantry	Lack of Awareness	210 (79.2)
	No time	5 (1.9)
	Not needed	29 (10.8)
	Live off campus	14 (5.3)
	Cook at home	2 (0.8)
	Not getting a reply	2 (0.8)
	Can’t cook	1 (0.4)
	No transportation	2 (0.8)
Knowledge about the Panther Meal share program?	Yes	86 (30.1)
	No	200 (69.9)
Do you use or have you ever benefited from the panther meal share program?	Yes	43 (15.0)
	No	243 (85.0)
Reason for non-usage of panther meal program	Lack of Awareness	200 (90.0)
	Not needed	10 (5.0)
	Does not enjoy food campus	1 (0.5)
	Live off campus	9 (4.5)
Food security category	Full/marginal Food Security	105 (36.5)
	Low Food Security	83 (28.8)
	Very low food security	100 (34.7)
Best channel to communicate Nutrition and health information?	Text messages	105 (36.8)
	Instagram	30 (10.5)
	Twitter	10 (3.5)
	Tik Tok	18 (6.3)
	WhatsApp	2 (0.7)
	Email	112 (39.3)
	Others (snapchat etc.)	8 (2.8)

**Fig. 1:**
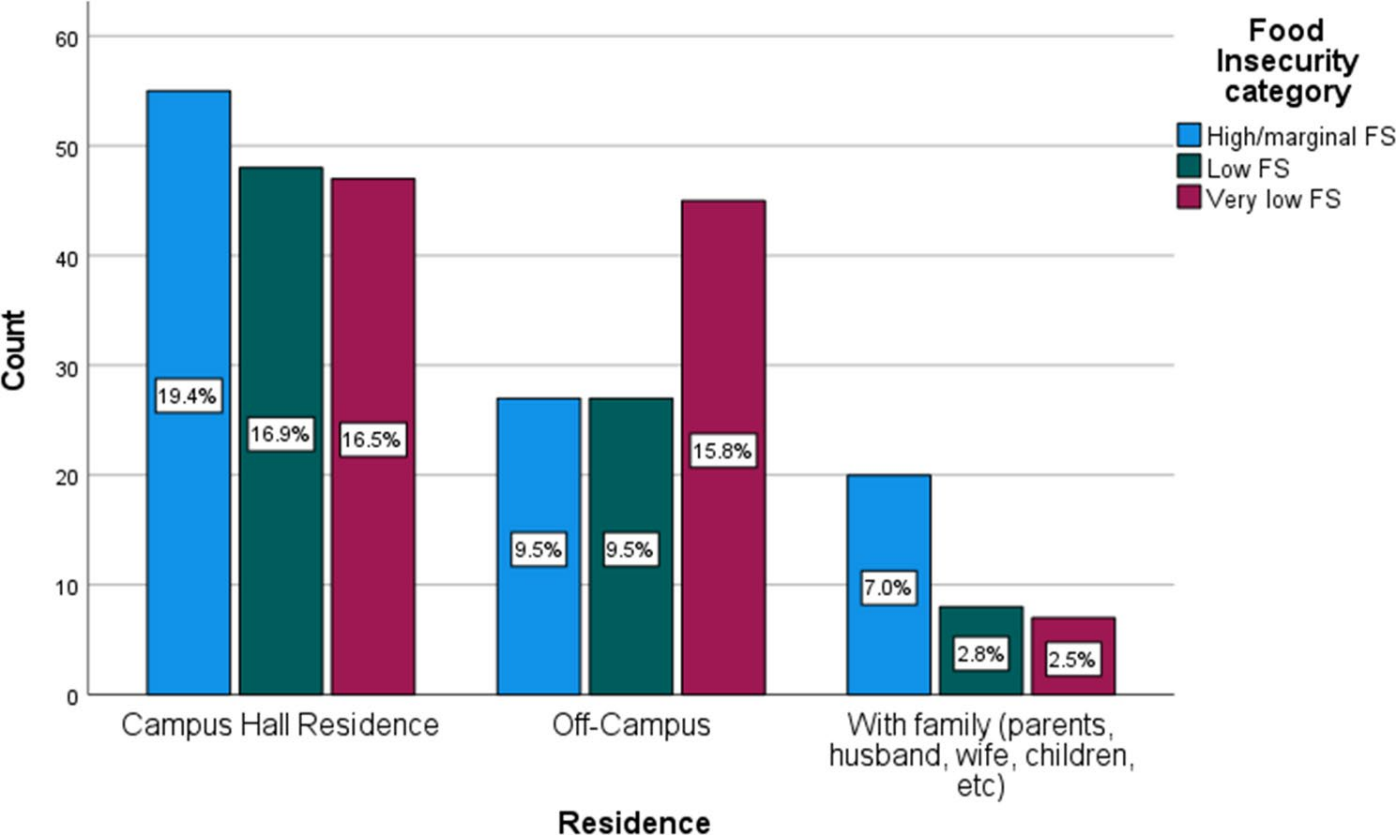
Food insecurity category by residence

**Fig. 2:**
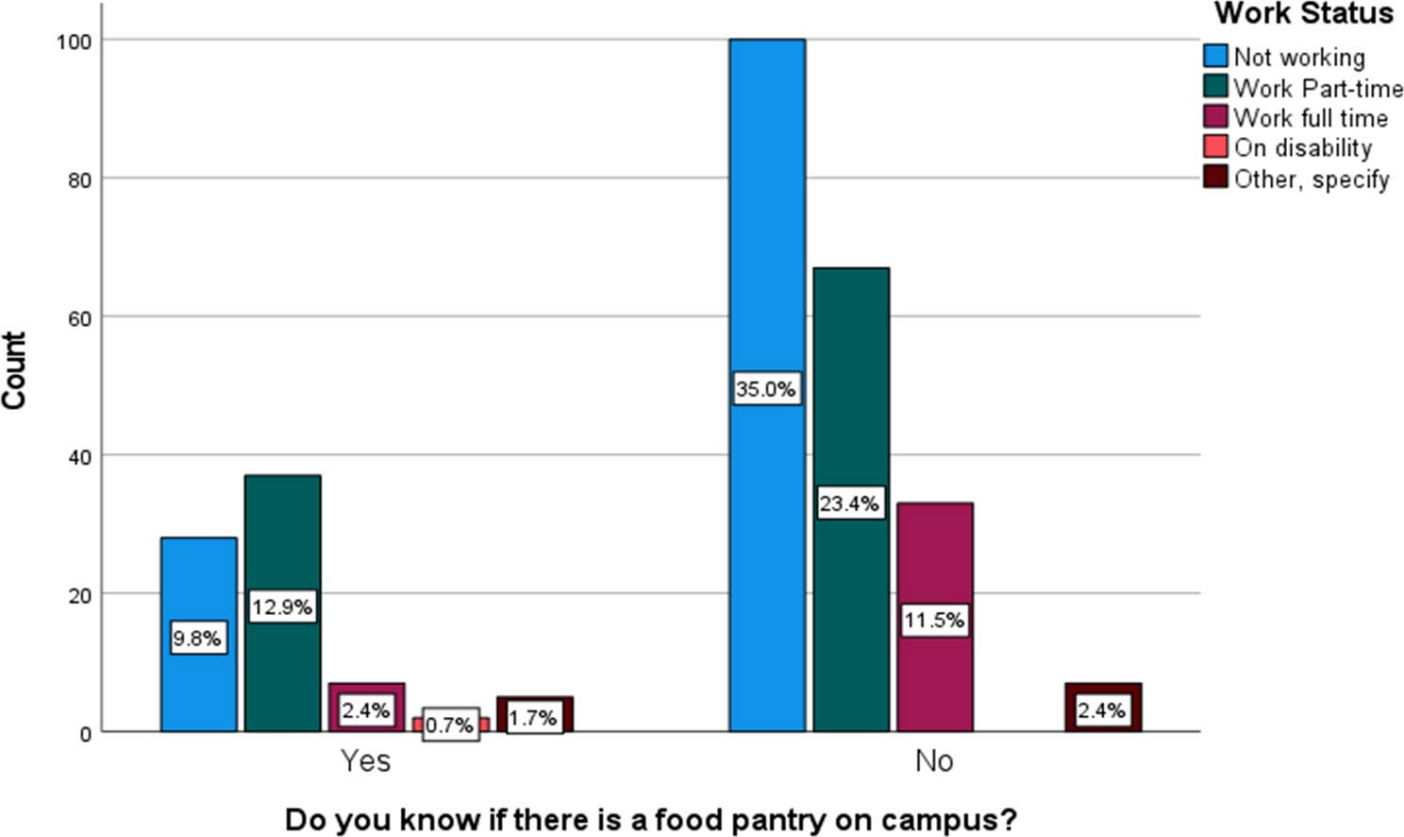
Awarness about food pantry by work status and classification

**Fig. 3:**
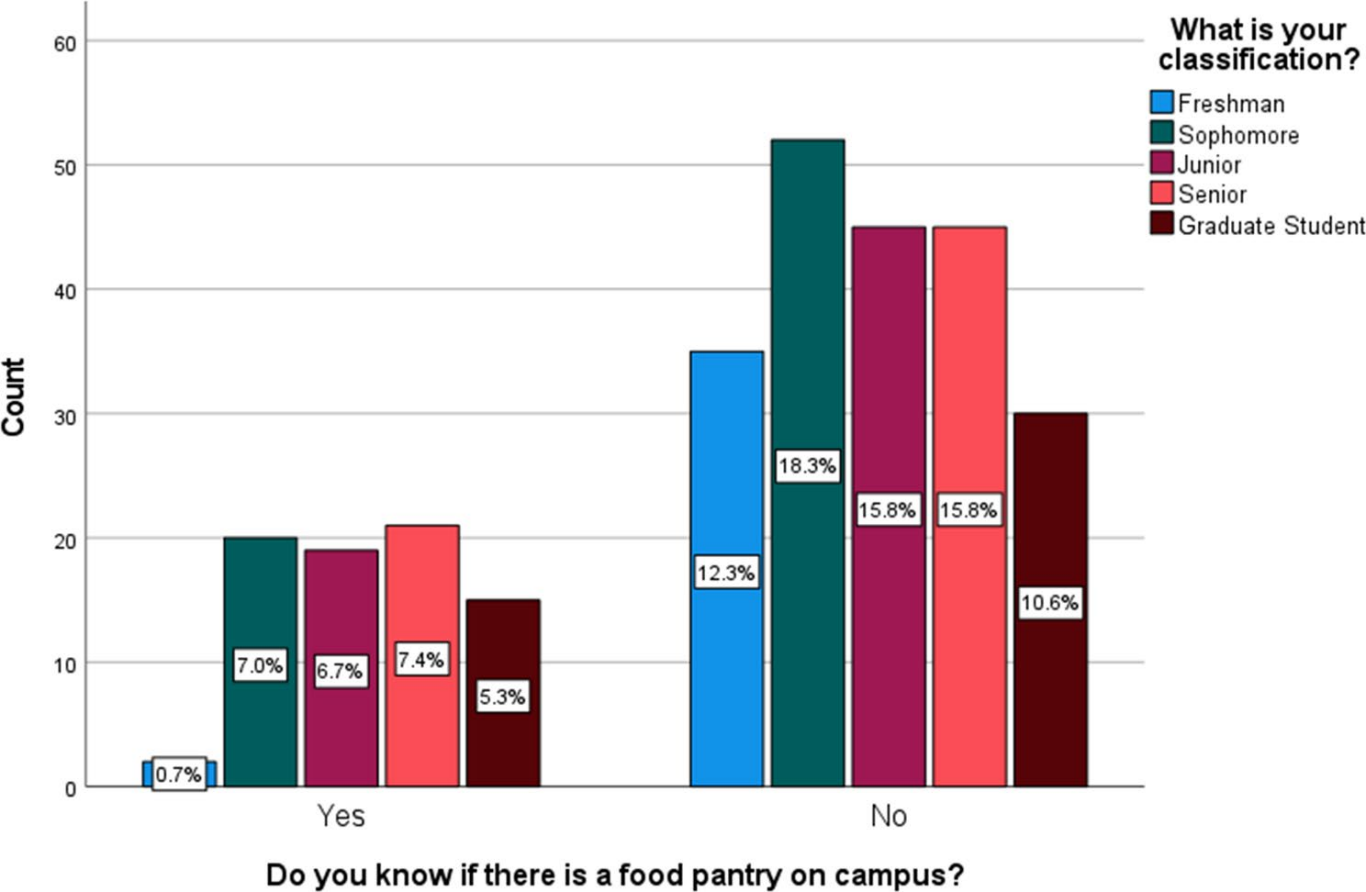
Awarness about foog pantry by classification

In Table 3, the analysis revealed a significant association between FI and the place of residence (*p* = 0.027). Figure 1 (FI category by residence) illustrates that individuals residing off-campus exhibit higher proportions of FI compared to residents of other housing arrangements. Awareness of the food pantry was significantly associated with work status (*p* = 0.008) and academic school year (*p* = 0.030) and with a larger proportion of those aware about the food pantry being those who work part-time and seniors, respectively (Figs. 2 and 3, Awareness about food pantry by work status & Awareness about food pantry by school year).

**Table 3:** Association of type of sociodemographic characteristics with awareness about campus resources and food insecurity (P-value)

	Awareness about food pantry	Awareness about panther meal share	Utilization of the food pantry	Utilization of panther meal share program	Food insecurity
Gender	0.641	0.096	0.083	0.508	0.209
Age	0.684	0.626	**0.017**	0.160	0.087
Ethnicity	0.744	0.684	0.264	0.394	0.070
Classification	0.**030**	0.889	0.060	0.421	0.706
College	0.815	0.078	0.637	0.065	0.748
Marital	0.356	0.724	0.511	0.891	0.899
Work status	**0.008**	**0.019**	0.119	0.199	0.187
Residence	0.779	0.547	**0.023**	**0.002**	0.**027**
Food insecurity	0.326	0.550	0.090	**0.034**	
Utilization of the food pantry	< 0.001	< 0.001			
Utilization of panther meal share	< 0.001	< 0.001			

Table 4 shows the results of two binary logistic regression models predicting awareness of the food pantry and utilization of the meal share program. Even though all the associated sociodemographic variables from Table 3 were initially put into the 4 models with awareness of food pantry, awareness of the meal share program, utilization of food pantry and utilization of the meal share program as outcomes, only significant predictors are shown on Table 4. Students’ school year was a predictor of the food pantry awareness. Freshmen, sophomores, juniors and seniors all had significant negative B coefficients and odds ratios less than 1 (*p* < 0.05). This implied that compared to graduate students, lower student school year levels have significantly lower odds of awareness of the campus food pantry while adjusting for other potential variables in the logistic regression model. Residence and FI status were predictors of the meal share program utilization. Compared to students residing in other types of residences, those living with family are significantly less likely to utilize the Panther Meal Share. Students living off-campus and in campus hall residences also show a trend towards lower utilization, but these results are less statistically significant. For example, students living with family have 71.8% lower odds (*p* = 0.001, OR = 0.28, 95% CI [0.132, 0.604]) of using the meal share program. Having high/marginal FS has significantly lower odds of using the meal share compared to low or very low FS (the reference). Odds are 64.3% lower for those with high/marginal FS (*p* = 0.033, OR = 0.37, 95% CI [0.148, 0.924]).

**Table 4:** Predictors of awareness and utilization of campus resources

Dependent Variable	Predictors	*P*-value	Odds ratio	95%CI Lower- upper
Awareness of food pantry	*Students’ Classification*			
	Graduate student	0.062		
	Senior	0.016	0.15	0.033–0.701
	Junior	0.009	0.13	0.028–0.596
	Sophomore	0.008	0.13	0.027–0.581
	Freshman	0.003	0.09	0.017–0.442
Utilization of meal share program	*Residence*			
	Others	0.006		
	With family	0.001	0.28	0.132–0.604
	Off campus	0.051	0.34	0.114–1.006
	Campus hall residence	0.074	0.07	0.004–1.296
	*Food insecurity*			
	Very low FS	0.065		
	Low FS	0.033	0.36	0.138–0.922
	High/marginal FS	0.033	0.37	0.148–0.924

## For the qualitative aspect of the study, five main themes emerged which include

### Poor awareness and visibility of campus resources

Both the quantitative and qualitative aspect of the study revealed a very poor awareness and utilization of the - food pantry and the meal share program. The awareness and utilization of the student led garden was explored through the qualitative study. The participants stated that most students don’t know about the campus resources and even some who have heard about them do not know where they are located or how to access them.

“*By percentage. I’ll say like 10% of students know about the food pantry. And then 5% of students know where it’s at. Because I didn’t even know the house had a basement till yesterday”.* – Student Key Informant.

*“The meal share program. Oh*,* I’ve heard of it before*,* but I don’t know*,* like*,* uh. what do they do?”* – Student Key Informant.

*“some students don’t know we have a student garden and some student*,* so it’s not that they’re not interested*,* they just might not know” –* Staff Key Informant.

### Bureaucratic process and logistics

Bureaucratic processes and logistical barriers hindered the use of the campus resources. The participant reported that complicated application processes and limited pickup times restricted access. Specific quotes are noted below as examples.

“*I wish there was a better way to plan it. You have to send them an email like*,* Hi*,* I’d like your inventory. And they’re like*,* okay*,* we’ll email you the list and you have to go edit the word document that they give you*,* email it back and then they’ll tell you*,* you can show up on this day and it’s like*,* I have class or am work and it’s just like*,* show up on this time or you don’t get the food at all* “ – FGD participant.

“*sometimes they’ve increased the requirement rules. So*,* we have more trainings and students don’t have to just sign a waiver*,* it’s more steps. Okay. And so that’s kind of tedious in a way” –*Staff key Informant.

### Insufficient funds, personnel and other resources to run the initiatives

This theme captured participants’ concerns about lacking the necessary funding, staffing, infrastructure, and operational capacity to successfully implement and sustain FS programs. As some participants described,

“*Lack of resources. That’s a big one…We have students who don’t have cars in our farm.” –* Student Key Informant.

*” We need labor force*,* things of that nature. Also*,* more work study or student working assistance” –* Staff Key Informant.

“*I think the biggest challenge is. It’s just having not having enough personnel to be able to sustain it… And I think the other thing is getting enough supplies*.*”* – Staff Key Informant.

### Ineffective communication

Some of the participants (stakeholders) complained about not knowing how to reach the students with information. Quotes from two of the participants are stated below.

“*Like I don’t know if my information doesn’t just get to them or what? But if I just e-mail or if I post flyers over there I don’t really have much engagement or interaction*,* but I have to like*,* go and ask for volunteers*,* for them who need volunteer hours. Maybe they can get to them. Can somebody come and assist me with this?”* – Staff Key Informant.

“*when you send the surveys out every month*,* every semester. We will still run into students who say*,* I did not know about it. And so how do we reach out to our students in helping them to know when we send it or why we send it to their student government?” –* Staff Key Informant.

Better marketing through text messages, social media, signage, and integration into new student orientation is needed.

*“I know that a lot of students*,* if they don’t use their university email*,* who use a preferred email. So*,* I think communication between the school and students will need to include preferred emails as well in order to increase that outreach.”* – FGD participant.

### Stigma due to location

This theme captures participants’ perspective that the campus food pantry being housed in the health center building deterred usage due to stigma. As one participant quoted,

“*I think that one of the worst things about some of the resources on campus*,* not just with the campus reserve*,* almost all of them*,* is that they’re in buildings that students never go into.”* –Student Key Informant.

Another participant explained further on how the health center location exacerbated stigma, stating

“*And then some there’s also a little bit of a stigma. You know*,* students see you going into that health center. A lot of students don’t like walking in there because they’re just thinking*,* Oh*,* you’re going in there because you’re mentally ill or you have an STD*.” – FGD participant.

The participant went on to elaborate that students may associate using the food pantry with poverty, saying

“*And then maybe*,* maybe the last minute they’ll think about the food pantry like*,* Oh*,* you’re going in there because you’re poor*,* right*?” – FGD participant.

In other words, locating the campus food pantry within the health center appeared to reinforce stigma and assumptions that discouraged some students from utilizing this resource. Participants felt moving it to a more central, accessible location could help mitigate this barrier. Reducing stigma represents an area for improvement in promoting FS initiatives.

## Discussion

The results of this mixed methods study illuminate significant barriers faced by students at a Texas HBCU, in accessing and utilizing campus FS resources. The results from both the quantitative and qualitative aspects of the study were integrated to complement and strengthen each other. The qualitative findings explained and added more depth to the quantitative results.

The quantitative findings showed limited awareness and use of existing programs like the food pantry and meal voucher system. Qualitative themes further shed light on how bureaucratic obstacles, resource constraints, communication breakdowns, stigma, and lack of understanding impeded students from taking full advantage of these initiatives. These challenges reflect broader systemic issues with implementing comprehensive FS programs at minority-serving institutions as barriers to wider adoption. A predominant finding highlighted the widespread lack of awareness and utilization of FS initiatives among students, even in the face of high rates of FI. From a quantitative perspective, only 8.1% of students reported utilizing the campus food pantry, and a mere 15% had accessed the meal voucher program. However, a significant 34.7% indicated experiencing very low FS, and 28.8% reported low FS, implying that 63.5% of the participants were food insecure. Students noted most of their peers were oblivious to options like the food pantry and community garden. This aligns with previous research finding that reported students at multiple universities failed to use campus food pantries even when facing FI, primarily because they did not know about them [14, 17, 18]. The regression model showed that as school year level goes lower from seniors to freshmen, the odds ratios decrease further below 1 showing progressively lower likelihoods of food pantry awareness. For example, the odds ratio for sophomores is 0.126. Meaning, sophomores have 0.126 times or 88.4% lower odds of awareness of the food pantry compared to graduate students.

Both the survey and interview data pointed to bureaucratic red tape as a hindrance to students accessing campus FS initiatives. Students described the process to use food pantries or apply for meal vouchers as convoluted, entailing extensive paperwork, documentation, and administrative steps. Prior research at community colleges found complex eligibility and application requirements posed barriers to students obtaining campus food assistance [19]. Inflexible scheduling of food pantry operating hours also made it difficult to visit around busy class schedules, a challenge noted in earlier studies [14, 18, 19]. Streamlining and standardizing processes could dismantle some bureaucratic obstacles to accessing resources. Simplifying enrollment and qualification processes may also minimize logistical obstacles that deter participation. Implementing user-friendly online portals for submitting documents, confirming eligibility, and scheduling food pantry visits could improve convenience. Extending pantry hours into evenings and weekends accommodates students with hectic schedules [14, 18]. Seeking regular user feedback helps modify procedures to ease new barriers as they emerge. Standard eligibility criteria and simplified intake procedures reduce administrative burdens. Raising awareness of available resources through targeted marketing and education is imperative to connecting food insecure students with initiatives that could alleviate their needs [20]. Strategically promoting services through social media campaigns, signage, and outreach at first-year orientation events could help bridge these information gaps.

The significant percentage of students experiencing very low FS underscores large unmet needs. Qualitative themes around inadequate resources conveyed campus difficulties in mustering the budget, staffing, infrastructure, volunteers, and supplies to run initiatives equal to the scale of student needs. Such resource limitations have been cited as key factors behind HBCUs’ struggles to implement more expansive student support services [8, 19, 21]. Minority-serving institutions often lack funding for staff and infrastructure specifically devoted to publicizing and providing food, health, and wellness programming [8, 22]. Securing dedicated operational funding, rather than depending on unstable grants and donations, provides stability to grow and sustain programs. Reliable budgets enable hiring permanent staff to manage initiatives, instead of overburdening faculty and volunteers. Investing in upgraded spaces and technologies expands capacity and efficiency. Exploring corporate sponsorships and community partnerships provides alternative funding channels [23]. Involving students in planning helps ensure initiatives remain aligned with evolving needs. Participants emphasized that communication lapses posed barriers to informing students about available food resources. They noted print flyers and emails frequently failed to reach students, who overlook these traditional outreach methods. Prior studies similarly found printed materials to have limited effectiveness for raising food pantry awareness [14, 17]. University emails often go ignored, signaling a need for enhanced approaches. Our study shows that other means of communicating may be effective, with 36.8% of the participants indicating that their preferred means of communication is via text messages. Student-university communication divides likely stem from differing cultural orientations between schools administrators vis-a-vis students expected to use services [13, 24]. Grassroots promotion through social media influencers, student groups, and cultural centers may better engage diverse student constituencies [25]. Fostering collaborations between staff and students embed initiatives within existing communities. Simplified messaging and branding focused on resonating with students can make communications more relatable and memorable [14, 26]. 

An unanticipated theme was the stigma introduced by housing the campus food pantry within the health center building. Students felt the location reinforced assumptions that using the pantry implied mental illness or poverty. Past studies found stigma to be a foremost barrier preventing college students from seeking food assistance [14, 17, 27]. The visibility and perceptions tied to facilities can either worsen or alleviate stigma. Relocating food pantries and services to more convenient, discreet, destigmatized locales could improve student engagement. Integrating them into spaces perceived positively like recreation centers mitigates stigma. Normalizing food assistance through supportive messaging also reduces stigma [13, 14, 27]. 

Another theme was students’ lack of understanding about the purpose and value of some campus FS initiatives. Participants gave examples of agriculture students not realizing the community garden aimed to provide nutritious food, or students being confused about payment options in the cafeteria. Unclear understanding about the intentions and functionality of resources acted as a barrier to more effective use. Targeted education through multiple channels can reinforce the benefits and aim of initiatives to boost participation. These results corroborate past evidence [7, 12, 24]. that systemic barriers impede HBCUs from delivering comprehensive FS programs.

The specific challenges around awareness, bureaucratic obstacles, constrained capacity, communication gaps, stigma, and lack of understanding found in this study echo prior works on hurdles faced in minority-serving institution contexts [17, 25–29]. The findings suggest tailored solutions like enhanced outreach, process simplification, discreet locations, anti-stigma messaging, and student partnership could help mitigate issues identified at this HBCU. Beyond specific barriers uncovered, our findings point to larger policy and practice changes needed to promote equitable food access in underserved college communities. Regular local and national surveys measuring campus FI are vital to track progress [30–32]. Ultimately, ensuring college students from all backgrounds have reliable access to nutritious food requires multifaceted responses spanning public policy, university programs, partnerships, and research. However, fully addressing FI requires comprehensive solutions beyond emergency food aid to provide sustainable, dignified access [33–35]. 

This study had limitations including a small sample size and single institutional focus. Additionally, the convenience sampling method used for the cross-sectional study means that the reported prevalence of FI may not accurately reflect the true prevalence on campus. The HBCU context allowed in-depth investigation of barriers within this population, but future studies could gather data from a diversity of institutions and larger samples. The self-reported nature of the survey data and variation in how different sub-populations perceive and experience FI should also be considered. Longitudinal data would provide richer insights into how students’ FS status and use of campus resources fluctuate over time.

## Conclusion

In conclusion, this study offers a snapshot of real barriers miring campus FS efforts at an HBCU, providing direction for policy and program responses. This contributes to the existing body of literature on FS by specifically examining the challenges faced by students within the unique context of HBCUs. The findings contribute valuable qualitative textures supplementing the quantitative prevalence data. Addressing these challenges requires a multifaceted approach that considers financial constraints, cultural considerations, stigma, awareness, institutional infrastructure, policy gaps, and the dynamic nature of student housing. By understanding these barriers, researchers and administrators can develop targeted strategies to enhance the effectiveness of FS initiatives in HBCUs and create a more supportive environment for student success. Findings from this research can inform the development of targeted strategies and policies to enhance the effectiveness of FS initiatives in HBCUs, ultimately promoting a healthier and more supportive environment for student success.

## Data Availability

“The datasets used and/or analyzed during the current study are available from the corresponding author on reasonable request”.
